# The Effect of Emodin-Assisted Early Enteral Nutrition on Severe Acute Pancreatitis and Secondary Hepatic Injury

**DOI:** 10.1155/2007/29638

**Published:** 2007-09-16

**Authors:** Gang Wang, Bei Sun, Yue Gao, Qing Hui Meng, Hong Chi Jiang

**Affiliations:** Department of Hepatobiliary Pancreatic Surgery, First Clinical Hospital, Harbin Medical University, 23 Youzheng Street, Nangang District, Harbin, Heilongjiang 150001, China

## Abstract

Severe acute pancreatitis (SAP) characterized by atrocious progression and numerous complications often leads to a high mortality rate due to hypermetabolism, systemic inflammatory response syndrome (SIRS), and multiple organs dysfunction syndrome (MODS). Studies have revealed that both early enteral nutrition (EEN) and emodin are potent agents in the management of SAP. However, whether the combined strategy is rational and more effective than either one alone remains unknown. In this regard, Wistar rats were treated with emodin-assisted EEN (EAEEN) through enteral nutrient tubes after induction of SAP by retrograde infusion of 5.0% sodium taurocholate into the common pancreatic duct. Serum levels of amylase, tumor necrosis factor-alpha (TNF-α), angiotensin II (AngII), maleic dialdehyde (MDA), glutamic pyruvic transaminase (ALT), glutamic oxaloacetic transaminase (AST) and C-reactive protein (CRP), intestinal secretory IgA (SIgA), pancreatic and hepatic myeloperoxidase (MPO) activity as well as plasma levels of D-lactate and endotoxin were measured. In addition, pathologic alterations of pancreas and liver were observed microscopically. We found that EAEEN could significantly ameliorate these parameters and prevent pancreas and liver from serious damage. In conclusion, Our results indicated that EAEEN could exert beneficial effects on experimental SAP and obviously abate the severity of secondary hepatic injury. The combined strategy was safe and more effective than either one alone in the acute stage of SAP. This study also provided an experimental base for the clinical treatment of SAP patients with EAEEN.

## 1. INTRODUCTION

Severe acute pancreatitis (SAP), characterized by atrocious progression, multicomplications, and difficult treatment [1–3] is an acute abdomen that ranges from a mild illness to a life-threatening condition. Clinically, the overall mortality rate for SAP is approximately 38.4% [4]. In spite of decades of intensive study, we are still in lack of etiological therapy. SAP causes such a hypermeolic state that adequate supply of nutrients plays a decisive role to reverse systemic malnutrition, potentiate resistance against infection, block the pathological deterioration, and facilitate recovery [5– 7]. In the past, it was believed that early enteral nutrition (EEN) in patients with SAP might exacerbate the clinical as well as pathological conditions and provoke serious complications. However, therapeutic strategies have changed dramatically over the past 20 years. The high mortality rate of SAP is often a result of multiple organ dysfunction syndrome (MODS) and appears to be especially related to systemic inflammatory response syndrome (SIRS) or even to infections of the necrotic pancreas. Since MODS and SIRS are apt to be facilitated with gut mucosal dysfunction and abundant Gram-negative bacteria as well as other gastrointestinal pathogens are commonly detected in pancreatic infections, the gut is considered to be the main source of SAP-related septic complications, so that maintaining gut barrier is equally as important as resting the pancreas when the inflammation within the gland resolves.

Though total parenteral nutrition (TPN) has been the standard practice for providing exogenous nutrients during the early stage of SAP, long-term administration of TPN can produce catheter-related complications, weaken systemic immunity, and induce secondary enterogenous infection. In comparison to TPN, EEN helps enhance the immunological and mechanical functions of intestinal mucosa, maintain the balance of gut microflora, reduce oxidative stress, prevent secondary infections, and improve therapeutic results. Emodin, the main effective component of rhubarb, which is a traditional Chinese herbal medicine and has the function of purgation, heat-clearing, and detoxicating as well as promoting blood circulation to remove blood stasis, has proved to be a beneficial pharmacy for SAP [8–12]. Because of the liver's special position in the gut circulation and the unsubstitutable role played in body metabolism, it becomes a major target for extrapancreatic damage of SAP. And liver injury, in turn, deteriorates SAP and amplifies the systemic damage caused by MODS. Therefore, effective prevention of liver injury is a key to shortening the course of SAP and decreasing the related mortality.

EEN and emodin have been fully affirmed by most scholars as an important part of therapy for SAP. However, it is still controversial what substance and dosage in EEN and what timing and route of administration of EEN associated with emodin are more appropriate and whether the combined treatment is more effective than either one alone. In the present experiment, we tried to explore possible mechanisms of EEN and emodin in the treatment of SAP as well as its secondary hepatic injury, and to demonstrate the feasibility and effectiveness of emodin-assisted early enteral nutrition (EAEEN).

## 2. MATERIALS AND METHODS

### 2.1. Materials

Sixty healthy male Wistar rats, weighing 190–250 g, were purchased from Animal Center, the First Clinical Hospital of Harbin Medical University (Harbin, China). Emodin was obtained from Pharmacy, the First Clinical Hospital of Harbin Medical University. Pepti-2000 variant was purchased from Nutricia (Zoetermeer, The Netherlands). Sodium taurocholate was obtained from Sigma (St. Louis, Mo, USA).

### 2.2. Animal models

Sixty male Wistar rats, housed in cages with a controlled temperature of 26°C and 12-hour light-dark cycles, were fed standard laboratory chow as well as water ad libitum, and allowed to acclimatize for at least a week. The rats were fasted overnight with free access to water before experiments. Surgical anesthesia was performed by intraperitoneal injection of 1% pentobarbital sodium (40 mg/kg body weight). SAP was induced by retrograde infusion of 5.0% sodium taurocholate (0.15 mL/100 g body weight) into the common pancreatic duct [13]. The “out” end of the enteral nutrient tube was placed at 7 cm from the far point of Treitz ligament, while the “in” end was dragged backward through the body wall and fixed on the back. The experimental protocol was undertaken in accordance with the guideline for the care and use of laboratory animals in research and was approved by the Ethical and Research Committee of the First Clinical Hospital, Harbin Medical University.

### 2.3. Experimental groups

Sixty rats were randomly divided into four groups (n=15) after SAP induction: control group (group A), emodin group (group B), EEN group (group C), and EAEEN group (group D). Control group was allowed free access to water without any intervention; Emodin group received emodin (3.0 mg/100 g body weight) through enteral nutrient tube at 12 h, 22 h, 32 h, 42 h, 52 h, and 62 h, respectively; EEN group received Pepti-2000 variant through enteral nutrient tube at 15 h, 25 h, 35 h, 45 h, 55 h, and 65 h, respectively. The enteral nutrient solution was continuously infused at each time point and the dosage at 15 h, 25 h, and 35 h was 120 mL/kg (body weight) followed by that increasing to 240 mL/kg (body weight) at 45 h, 55 h, and 65 h; EAEEN group received emodin (3.0 mg/100 g body weight) at 12 h, 22 h, 32 h, 42 h, 52 h, and 62 h and Pepti-2000 variant at 15 h, 25 h, 35 h, 45 h, 55 h, and 65 h. The dosage and infusion method of Pepti-2000 variant at each time point in EAEEN group were the same as those in EEN group. Both emodin and the enteral nutrient solution were given by infusion pump.

The remaining living animals in each group were reanesthetized 72 hours after SAP induction using intraperitoneal injection of 1% pentobarbital sodium (40 mg/kg body weight). Laparotomies were performed and samples of blood and tissues were immediately obtained.

### 2.4. Histopathologic examination

A portion of the pancreatic tail and hepatic and ileal tissues from each rat was incised and the pancreatic as well as hepatic tissues were fixed in 10% neutral-buffered formalin, embedded in paraffin, and stained with hematoxylin and eosin (HE) for light microscopy. Two experienced pathologists who were blinded to the experimental protocol scored the pancreatic tissue on a scale from 0 to 4 for the degrees of edema, inflammation, hemorrhage, and necrosis in 20 fields. We applied the scoring system defined by Kusske et al. [14] and the scores of each histologic examination were totaled. The ileal tissue was observed to measure the villus height of ileum microscopically.

### 2.5. Secretory IgA (SIgA), myeloperoxidase (MPO) activity, and ascitic fluid

The jejunum and ileum were excised and the intestinal mucus was collected to measure the level of SIgA by radio immunoassay with the kit (Institute of Atomic Energy Physics, Chinese Academy of Sciences, China). The values of MPO activity in pancreas and liver were measured to assess polymorphonuclear leukocyte (PMN) infiltration. A portion of the pancreatic and hepatic tissues was obtained to determine pancreatic and hepatic MPO activity by chromatometry with the kit (Invitrogen, Calif, USA) and ascitic fluid was quantified.

### 2.6. Edema

After removal, fresh samples of pancreas and ileum were immediately weighed and dried at 100°C for 24 hours and reweighed. The wet-dry weight ratio of pancreas (pww/dw) and ileum (iww/dw) were calculated to determine the degrees of pancreatic edema and ileal edema.

### 2.7. Laboratory test

Blood samples were collected from abdominal aorta and centrifuged at 3000 rpm for 5 minutes. The serum was captured and stored at −20°C until assayed. Tumor necrosis factor-alpha (TNF-α) and angiotensin II (AngII) were measured by radio immunoassay with the kits (Institute of Atomic Energy Physics, Chinese Academy of Sciences, China). Maleic dialdehyde (MDA), C-reactive protein (CRP) (Jiancheng Biotech Ltd., Nanjing, China), and plasma D-lactate (Sigma, USA) were assessed by enzymatic-spectrophotometric method. Plasma endotoxin (Shanghai Med & Chem Institute, Shanghai, China) was tested by chromogenic limulus amebocyte lysate technique. Glutamic-pyruvic transaminase (ALT), glutamic-oxaloacetic transaminase (AST), and amylase were determined by automated HITACHI-7150 analyzer.

### 2.8. Statistical analysis

Results were expressed as mean ± standard deviation (SD). The significance of differences in histopathologic scores was assessed by Kruskal-Wallis test. Other continuous data were analyzed by factorial design ANOVA test. Statistical analysis was finished by SPSS10.0 statistical program and difference was considered statistically significant when P<.05.

## 3. RESULTS

**Figure 1:**
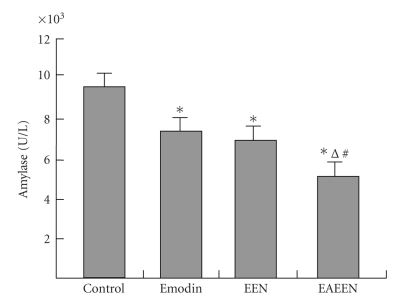
Changes of serum amylase levels in different groups. *P<.05 versus control group; ^Δ^ P<.05, EAEEN group versus Emodin group; ^*#*^ P<.05, EAEEN group versus EEN group. EEN: early enteral nutrition. EAEEN: emodin-assisted early enteral nutrition.

**Figure 2:**
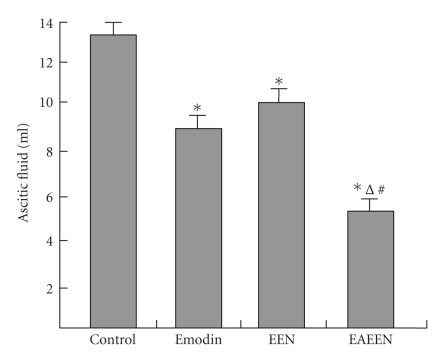
Changes of the amount of ascitic fluid in different groups. *P<.05 versus control group; ^Δ^ P<.05, EAEEN group versus Emodin group; ^*#*^ P<.05, EAEEN group versus EEN group. EEN: early enteral nutrition. EAEEN: emodin-assisted early enteral nutrition.

**Figure 3:**
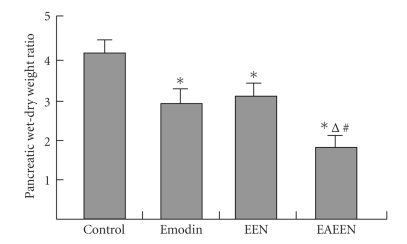
Changes of pancreatic wet-dry weight ratio in different groups. *P<.05 versus control group; ^Δ^ P<.05, EAEEN group versus Emodin group; ^*#*^ P<.05, EAEEN group versus EEN group. EEN: early enteral nutrition. EAEEN: emodin-assisted early enteral nutrition.

**Figure 4:**
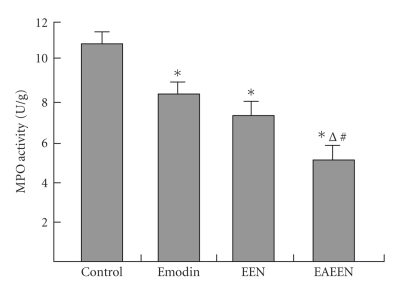
Changes of MPO activity of pancreas in different groups. *P<.05 versus control group; ^Δ^ P<.05, EAEEN group versus Emodin group; ^*#*^ P<.05, EAEEN group versus EEN group. EEN: early enteral nutrition. EAEEN: emodin-assisted early enteral nutrition. MPO: myeloperoxidase.

**Figure 5:**
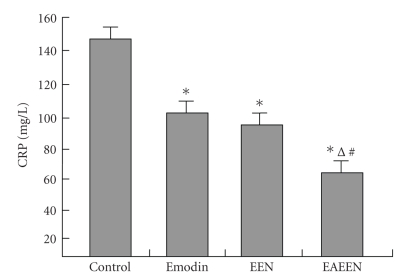
Changes of serum CRP levels in different groups. *P<.05 versus control group; ^Δ^ P<.05, EAEEN group versus Emodin group; ^*#*^ P<.05, EAEEN group versus EEN group. EEN: early enteral nutrition. EAEEN: emodin-assisted early enteral nutrition. CRP: C-reactive protein.

Before sampling 72 hours after the induction of SAP, the ratio of survival to death was 7 to 8 in control group, 9 to 6 in emodin group, 8 to 7 in EEN group, and 12 to 3 in EAEEN group. Compared with control group, there were significant differences in the following data as described in emodin, EEN, and EAEEN groups (P<.05, Tables 1–4, Figures 1–5).

### 3.1. Macroscopic findings

In group A, severe edema, diffuse hemorrhage, and necrosis in pancreas were observed. The gastrointestinal tract was obviously ectatic and edematous. There was much bloody ascites in the peritoneal cavity. Compared with group A, the severity of macroscopic changes was significantly abated in groups B, C, and D.

### 3.2. Laboratory test

In contrast to emodin and EEN, EAEEN significantly reduced the levels of TNF-α, AngII, MDA, ALT, AST, CRP, and amylase in serum as well as the levels of plasma endotoxin and D-lactate (P<.05, Tables 1, 2, and 4, Figures 1 and 5). Differences in these data were not considered statistically significant between group B and group C.

### 3.3. Edema, SIgA, MPO, and ascitic fluid

The levels of MPO in pancreas and liver, pww/dw, iww/dw, and the amount of ascitic fluid were significantly lower, while the level of SIgA was obviously higher in group D than those in group B and group C (P<.05, Tables 2, 3, and 4, Figures 2–4). However, no significant differences were found in these data between group B and group C.

### 3.4. Histopathologic examination

**Figure 6:**
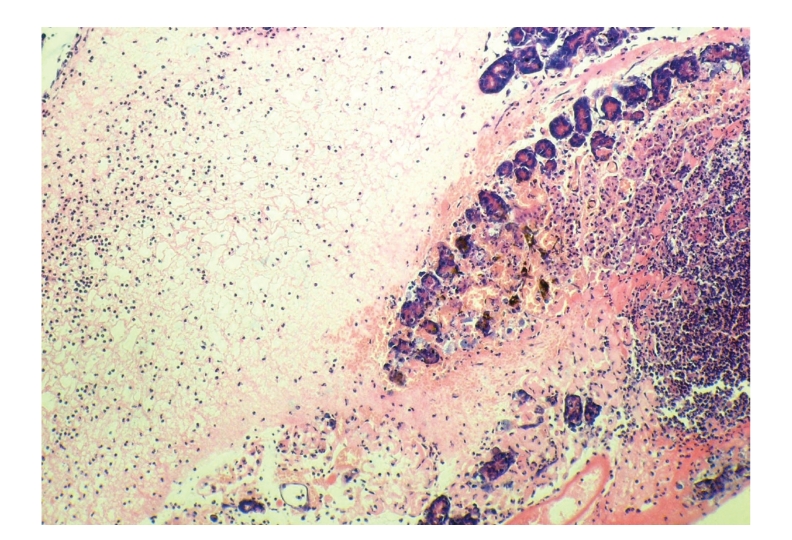
Histopathologic sections of pancreas (HE, x100), A: control group. The pancreas of control group showed extensive necrosis, diffuse edema, and massive inflammatory cells infiltration.

**Figure 7:**
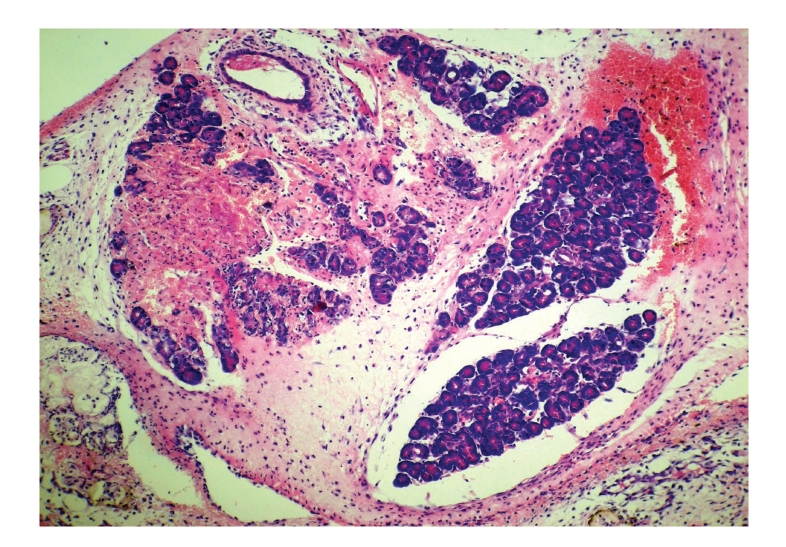
Histopathologic sections of pancreas (HE, x100), B: emodin group. The pancreas of emodin group showed moderate reduction in necrosis, edema, and inflammatory cells infiltration as compared with those in control group.

**Figure 8:**
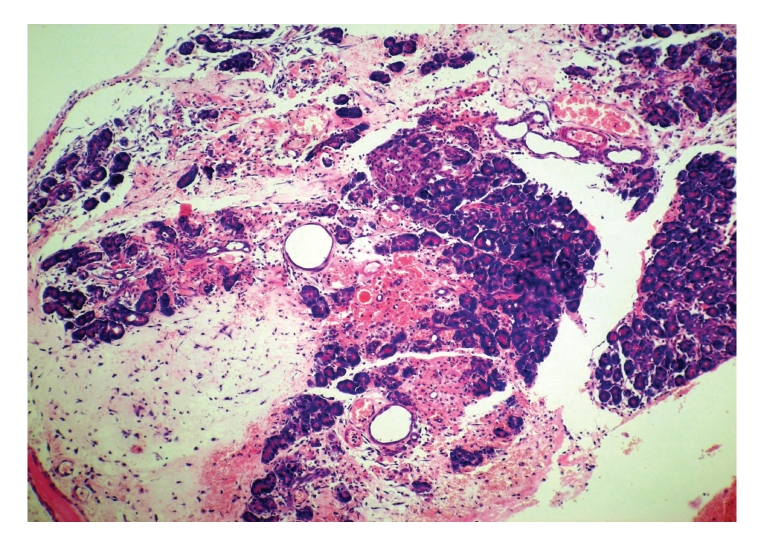
Histopathologic sections of pancreas (HE, x100), C: EEN group. The pancreas of EEN group also showed moderate reduction in necrosis, edema, and inflammatory cells infiltration as compared with those in control group. EEN: early enteral nutrition.

**Figure 9:**
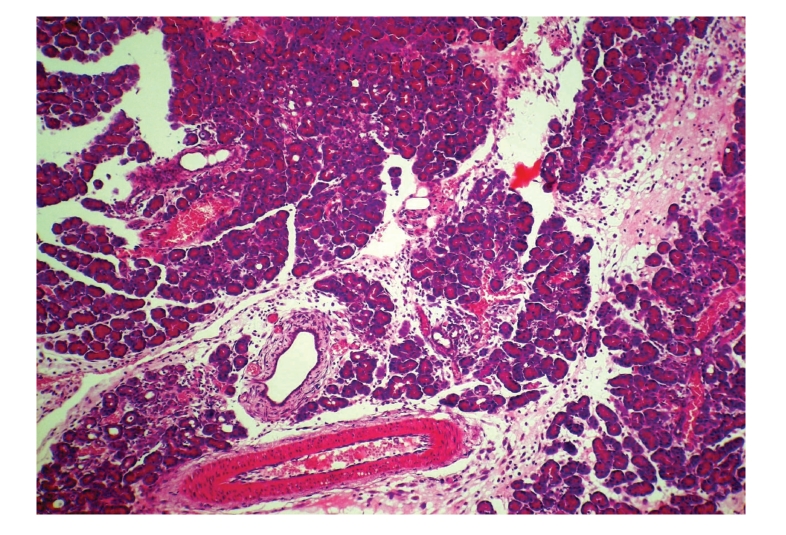
Histopathologic sections of pancreas (HE, x100), D: EAEEN group. The pancreas of EAEEN group showed significant reduction in necrosis, edema, and inflammatory cells infiltration as compared with those in control group, EEN group and emodin group. EAEEN: emodin-assisted early enteral nutrition EEN: early enteral nutrition.

**Figure 10:**
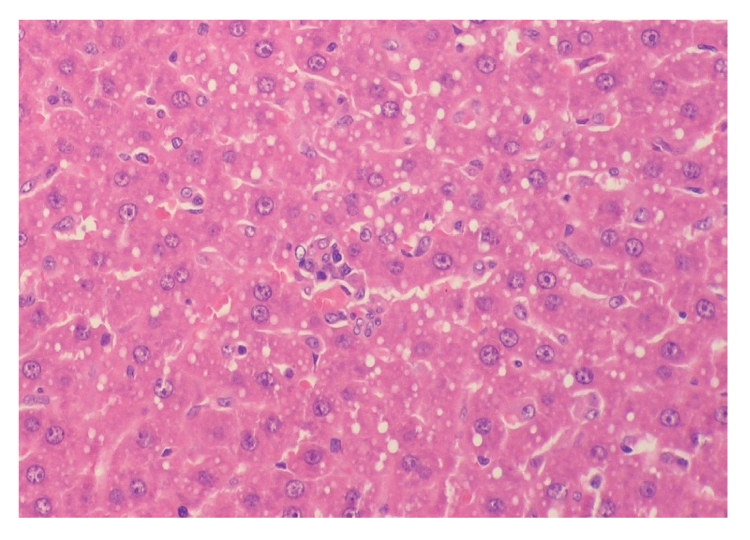
Histopathologic sections of liver (HE, x200), A: control group.

**Figure 11:**
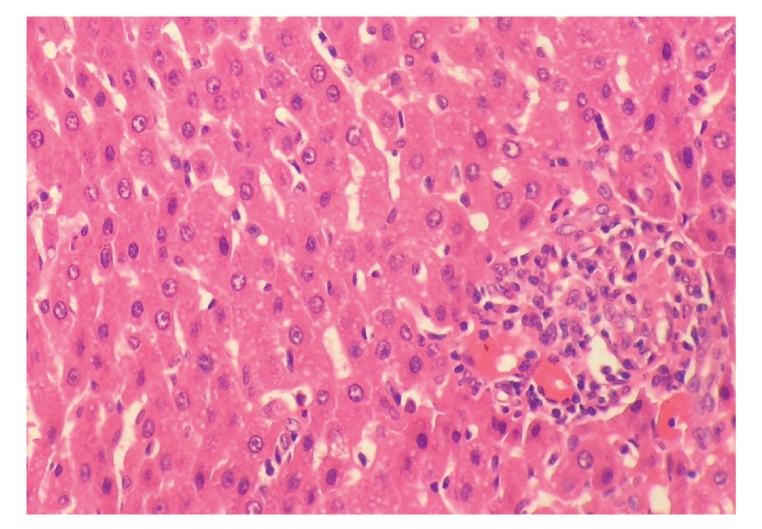
Histopathologic sections of liver (HE, x200), A: control group. Figures 10 and 11 showed many inflammatory cells infiltration in portal area, hepatic endothelial cells hyperplasia, vacuolar degeneration, and punctate necrosis of hepatic cells in control group.

**Figure 12:**
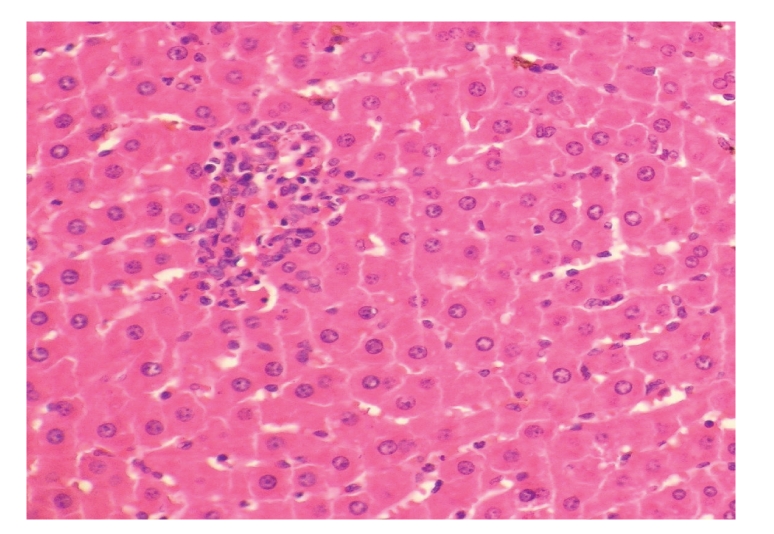
Histopathologic sections of liver (HE, x200), B: emodin group. The liver of emodin group showed few inflammatory cells infiltration in portal area and there were no obvious changes in hepatic cells.

**Figure 13:**
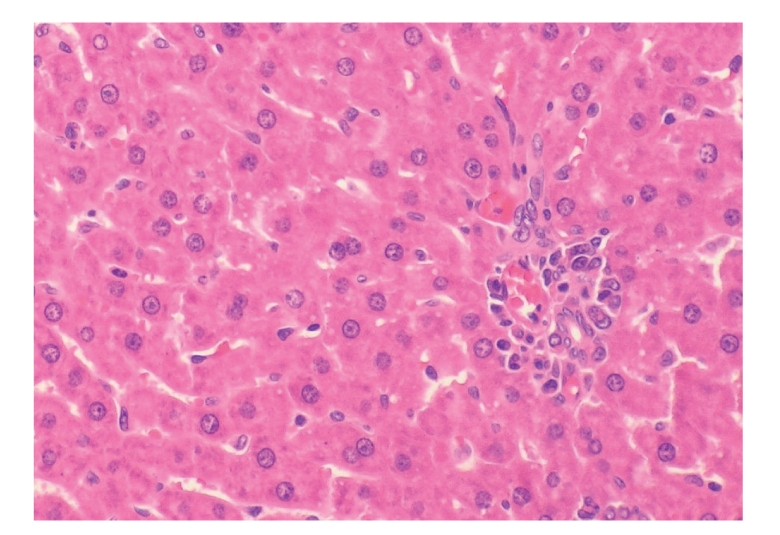
Histopathologic sections of liver (HE, x200), C: EEN group. The liver of EEN group also showed few inflammatory cells infiltration in portal area and the hepatic cells indicated no obvious alterations.

**Figure 14:**
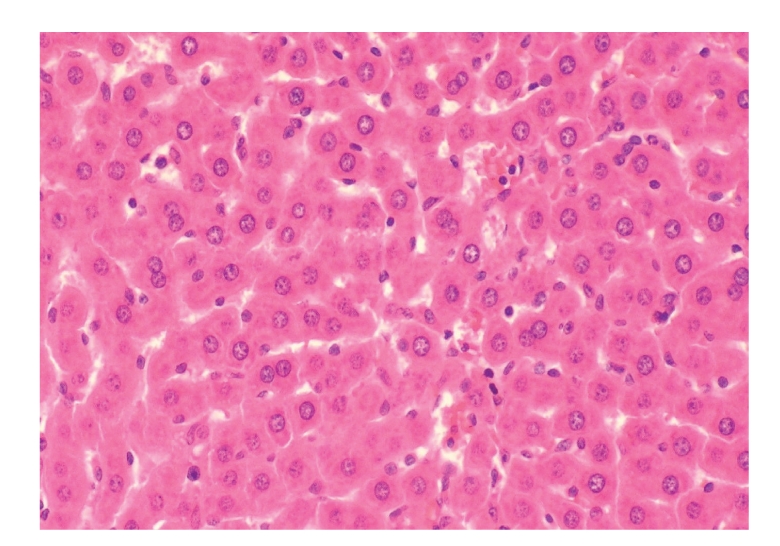
Histopathologic sections of liver (HE, x200), D: EAEEN group. The hepatic cells showed no significant changes and the hepatic lobules were nearly in normal states in EAEEN group.

The degrees of edema, inflammatory infiltration, hemorrhage, and necrosis and the scores of histopathologic alterations in pancreas were significantly reduced in group D as compared with those in group B and group C (P<.05, Table 3, Figures 6–9). EAEEN could also attenuate the severity of secondary liver injury obviously (Figures 10–14). The villus height of ileum was significantly increased by EAEEN compared with emodin and EEN (P<.05, Table 3). There were no significant differences in the scores of pancreatic histopathologic alterations as well as the ileal villus height between group B and group C.

## 4. DISCUSSION

Emodin characterized by the main active monomer of rhubarb is a derivative of anthraquinone (3-methyl-1, 6, 8-trihydroxyanthraquinone). Studies have proved that emodin is a potent agent in the management of clinical and experimental acute pancreatitis (AP) [8]. Zhang et al. [9], and Wu et al. [12] found emodin had significant therapeutic effects on SAP rats by correcting intestinal flora disturbances, promoting intestinal peristalsis, inhibiting inflammatory cytokine release and pancreatin activity, and scavenging oxygen free radicals (OFR). Wu et al. [10] proved the mechanisms of emodin in the treatment of SAP included modulation of abnormal eicosanoid metabolism, promotion of pancreatic cytoprotection, and improvement of pancreatic microcirculation. Kumar et al. [11] found emodin was a potent inhibitor of nuclear factor-kappaB (NF-κB) activation as well as expression of adhesion molecules and could be useful in treating various inflammatory diseases. In a word, emodin has multiple beneficial effects on SAP.

In our experiment, administration of EAEEN could significantly abate the severity of SAP as compared with other groups, which was reflected by the reductions in levels of serum amylase, the amount of ascitic fluid, pancreatic MPO activity, pww/dw, and the pathologic scores of pancreas. Our results indicated that the advantages of EAEEN for the treatment of SAP existed in (1) EEN alone could enhance gut immunity, promote hyperplasia of intestinal mucosa, improve microcirculation of intestinal mucosa, decrease the permeability of intestinal mucosa, and reduce the incidence of translocation of intestinal bacteria; (2) Pepti-2000 variant, which is an elemental diet with low-fat content and can be absorbed easily, contains adequate nutritional ingredients, scanty residue and has low viscosity. Administration of Pepti-2000 variant by enteral nutrient tube at different time points conformed to the daily dietary habit, placed the pancreas at a “full rest,” and inhibited “rebound” of the inflammatory reaction; (3) emodin could clear the intestinal bacteria and toxin, promote intestinal peristalsis and recovery of the intestinal function, so as to reduce the incidence of secondary enterogenous infection. Taken together, this combined strategy was methodologically more rational and purposive, with undoubtedly higher efficacy.

A vicious cycle of pancreatic microcirculatory changes such as capillary stasis and vasoconstriction has been shown to occur in the course of SAP [15]. Renin-angiotensin system (RAS) is an important stress system in pancreatic microcirculatory disturbances. RAS in systemic circulation is activated to a great extent and produces massive angiotensinII (AngII), which subsequently combines with its receptors predominantly localized to the epithelia of pancreatic ducts, vascular endothelia, and pancreatic acinar cells [16], results in the strong vasoconstriction of pancreas, and aggravates the pancreatic ischemia and necrosis in SAP. In this study, our results proved that administration of EAEEN enhanced colloid osmotic pressure, prevented excessive peripancreatic and retroperitoneal fluid exudation followed by hypovolemic shock, and inhibited the activation of pancreatic enzymes [9, 12] so as to eliminate the main factors that induced the abnormal activation of RAS, further significantly reduced the level of serum AngII and ameliorated pancreatic microcirculatory disturbances.

Gut barrier dysfunction and intestinal immunosuppression are directly related to the severity of SAP, so that how to protect the intestinal mucosa from being damaged might play a key role in preventing bacterial and toxin translocation and deterioration of SAP. D-lactate is the indigenous metabolic product of intestinal resident flora. Normally, the level of blood D-lactate is quite low and the measurement of the increased plasma D-lactate level may be a useful marker to assess the intestinal injury [17]. SIgA, the predominant immunoglobulin present in mucosal secretions, is the major ingredient of the intestinal immunological function on mucosal surfaces. In this study, the levels of intestinal SIgA, plasma D-lactate, the ileal villus height, and iww/dw showed obvious differences in EAEEN group as compared with those in other groups. Our data demonstrated that administration of EEN associated with emodin could significantly attenuate the severity of gut damage and enhance the intestinal immunity by restoring the microecology of the intestinal flora, maintaining the intestinal epithelia integrity, abating edema of the intestinal wall, and promoting the secretion of intestinal immune substances so as to reduce the incidences of enterogenous infection and endotoxemia.

In SAP, bacterial and endotoxin translocation through the damaged gut barrier is the culprit for the development of secondary septic complications and a second peak of mortality. Endotoxin is the lipopolysaccharide (LPS) present in the Gram-negative bacterial wall. Abundant plasma endotoxin intensively remotivates mononuclear-macrophage system and accelerates the production of many cytokines as well as inflammatory mediators, which induce septic shock and amplify SIRS followed by the development of MODS after releasing into the blood. Atrociously amplified SIRS is a hallmark for SAP with elevated circulating proinflammatory cytokines including tumor necrosis factor-*α* (TNF-α) and interleukin-1. TNF-α is the most prominent “first-line” cytokine in SIRS and plays a central role in the pathogenesis of AP and related systemic complications [18]. TNF-α can induce infiltration and activation of neutrophils, upregulate cellular adhesion molecules, promote the secretion of OFR, initiate and aggravate the cascade of other proinflammatory cytokines and inflammatory mediators. MPO, a member of peroxidases secreted by neutrophils, is an indicator for the count and activity of neutrophils.

In our study, administration of EAEEN significantly reduced the levels of plasma endotoxin, serum TNF-α, and MPO in pancreatic tissue, so as to inhibit both local and systemic inflammatory reaction as well as oxidizing reaction and abate the severity of pancreatic damage. We enumerated the following explanations as the rationales: (1) emodin could inhibit the overgrowth of pathogenic organisms including Gram-negative bacteria in the intestinal tract [9] and accelerate their excretion by promoting intestinal peristalsis, so that the direct source of endotoxin was eradicated and the level of plasma endotoxin depressed; (2) EEN could potentiate plasma protein synthesis, increase the blood perfusion in portal system, promote hepatoenteral circulation, and enhance hepatic detoxification, which in turn greatly reduced the level of plasma endotoxin; (3) EEN improved the gut barrier function and maintained the balance of gut microflora so as to decrease the production of endotoxin and to prevent it from entering the blood, which consequently abated the remotivation of mononuclear-macrophage system, so that the releases of TNF-α and other cytokines were reduced; (4) emodin inhibited the activation of pancreatic enzyme [9] so as to prevent mononuclear-macrophage system, activated by pancreatic enzyme after its entering the plasma, from producing TNF-α; (5) AngII is capable of promoting the releases of proinflammatory cytokines and stimulating the migration and infiltration of monocytes and neutrophils [15, 19, 20]. Administration of EAEEN significantly reduced the level of serum AngII, which further decreased the concentration of serum TNF-α and pancreatic MPO activity so as to attenuate the severity of inflammation in pancreas; (6) TNF-α induces aggregation and activation of neutrophils in the pancreas. Administration of EAEEN could significantly reduce blood TNF-α level, which consequently reversed this process and abated the pancreatic inflammatory reaction.

Secondary liver injury (SLI) has a remarkable incidence in SAP, and the severity of hepatocyte injury is proportional to the severity of SAP. Several mechanisms are involved in the initiation of SLI: (1) in the acute stage, massive proinflammatory factors excessively activate the liver Kupffer cells, which then intensely release even more profactors, so that a cascade amplifying cycle inside and outside the liver is formed, bringing heavy inflammatory injury to the liver; (2) TNF-α and endotoxin induce massive neutrophils infiltration and activation, producing great amount of OFRs, which initiate intensive lipid peroxidations aggravating liver injury; (3) intestinal mucosal damage leads to a remarkable increase of endotoxin level in the portal circulation bringing enormous damage to the liver. Sameshima et al. [21] found that LPS could significantly elevate serum TNF-α activity and induce more severe liver damage in AP rats, which suggested that both LPS and TNF-α played critical roles in the development of secondary hepatic injury during AP. Tadao et al. [22] found that activated neutrophils and macrophages produced large amount of OFRs in SAP and they were closely involved in the secondary liver disease. MDA is the product of lipid peroxidation of OFR, which indicates the amount of OFR and indirectly reflects the severity of tissue damage. CRP is an acute phase reactive protein produced by the liver especially in the context of SAP. The increase of serum CRP is proportional to the severity of hepatocyte injury. Our experiment demonstrated that EAEEN could remarkably decrease TNF-α, MDA, and endotoxin levels in the blood and MPO activity in the liver, so that protect the liver from the tremendous inflammatory and oxidizing injuries brought upon by SAP, as manifested by the decreases of ALT, AST, and CRP levels in the blood.

In a word, administration of emodin prior to EEN formed a firm base for subsequent EEN therapy of SAP by clearing bacteria and toxin, promoting exsufflation and defecation, abating abdominal distension and systemic inflammatory reaction, so that the enteral nutrient solution could be fully absorbed. Furthermore, EEN could “compensate for” the massive loss of nutrients caused by administration of emodin, so they two could benefit each other in combination and obviously improve the therapeutic efficacy. In this experiment, administration of emodin and EEN in the early stage of SAP was safe and rational, and the combined strategy was more effective than either one alone. Our results showed that administration of EAEEN could control multiple deteriorating factors of SAP and provided an effective treatment for SAP and its secondary hepatic injury by protecting intestinal mucosal barrier, enhancing body immune competence, inhibiting systemic inflammatory reaction and reversing microcirculatory dysfunction, and so forth. This study suggested a broad potential value for EAEEN in the clinical treatment of SAP. However, further studies are needed to determine whether administration of EAEEN can also protect other organs from being damaged in SAP.

## Figures and Tables

**Table 1:** Levels of MDA, TNF-α, and AngII in 4 groups (EEN: early enteral nutrition. EAEEN: emodin-assisted early enteral nutrition. MDA: maleic dialdehyde. TNF-α: tumor necrosis factor-alpha. AngII: angiotensinII).

Groups	n	MDA (nmol/mL)	TNF-α (ng/mL)	AngII (pg/mL)
Control	15	13.63±0.19	2.27±0.41	287.93±54.39
Emodin	15	10.15±1.96*	1.88±0.25*	200.63±34.57*
EEN	15	9.94±0.82*	1.72±0.36*	219.77±68.79*
EAEEN	15	6.52±1.07*^Δ*#*^	1.38±0.17*^Δ*#*^	159.73±82.85*^Δ*#*^

*P<.05, versus control group; ^Δ^ P<.05, EAEEN group versus emodin group; ^*#*^ P<.05, EAEEN group versus EEN group.

**Table 2:** Levels of endotoxin, iww/dw, and D-lactate in 4 groups (EEN: early enteral nutrition. EAEEN: emodin-assisted early enteral nutrition. iww/dw: wet-dry weight ratio of ileum).

Groups	n	Endotoxin(EU/mL)	iww/dw	D-lactate(mmol/L)
Control	15	0.385±0.026	3.97±0.44	0.574±0.178
Emodin	15	0.271±0.015*	2.80±0.76*	0.417±0.014*
EEN	15	0.232±0.037*	2.73±0.19*	0.391±0.088*
EAEEN	15	0.114±0.048*^Δ*#*^	1.71±0.52*^Δ*#*^	0.282±0.009*^Δ*#*^

*P<.05, versus control group; ^Δ^ P<.05, EAEEN group versus emodin group; ^*#*^ P<.05, EAEEN group versus EEN group.

**Table 3:** The level of SIgA, the ileal villus height, and the pathologic scores of pancreas in 4 groups (EEN: early enteral nutrition. EAEEN: emodin-assisted early enteral nutrition. SIgA: secretory IgA).

Groups	n	SIgA(ug/mL)	The villus height of ileum(um)	The pathologic scores
Control	15	75.44±20.27	299.74±45.27	14.13±0.62
Emodin	15	120.83±32.21*	438.26±33.79*	10.38±0.54*
EEN	15	111.91±34.70*	446.39±60.71*	11.05±0.16*
EAEEN	15	161.86±19.90*^Δ*#*^	568.53±66.18*^Δ*#*^	7.19±0.43*^Δ*#*^

*P<.05, versus control group; ^Δ^ P<.05, EAEEN group versus emodin group; ^*#*^ P<.05, EAEEN group versus EEN group.

**Table 4:** Levels of ALT, AST, and hepatic MPO activity in 4 groups (EEN: early enteral nutrition. EAEEN: emodin-assisted early enteral nutrition. ALT: glutamic pyruvic transaminase. AST: glutamic oxaloacetic transaminase. MPO: myeloperoxidase).

Groups	n	ALT(U/L)	AST(U/L)	MPO(U/g)
Control	15	217.49±36.11	313.08±45.19	8.39±1.42
Emodin	15	177.64±54.30*	271.56±25.98*	6.24±1.34*
EEN	15	170.29±24.57*	259.23±53.77*	6.09±0.95*
EAEEN	15	90.95±55.67*^Δ*#*^	203.12±36.92*^Δ*#*^	3.95±0.71*^Δ*#*^

*P<.05, versus control group; ^Δ^ P<.05, EAEEN group versus emodin group; ^*#*^ P<.05, EAEEN group versus EEN group.
